# Dr. Henderson's Inquiry Respecting Dr. Milward

**Published:** 1811-12

**Authors:** A. Henderson

**Affiliations:** Golden Square


*To the Editors of the Medical and Physical Journal.*

GENTLEMEN,

TOWARDS the middle of the last century, "*A circular invitatory Letter to all Orders of Learned Men, concerning an Attempt or Essay towards an History of the Lives, Deaths,*

*Deaths, Writings, Characters and Opinions of the most celebrated British Physical and Chirurgical Authors, &c.*" was published by Dr. Edward Milward; and from the language of the author it may be inferred that he had made some progress in the undertaking, for which he thus solicited assistance: but the proposed work never appeared. Will you permit me to inquire, through the medium of your Journal, if any one can inform me how far he proceeded in his labour; and whether his papers are yet in being?

I remain, Gentlemen,

Your most obedient Servant,

A. HENDERSON.

*Golden Square, Nov.* 15, 1811.

